# Advances and Perspectives on the Epidemiology of Bovine *Cryptosporidium* in China in the Past 30 Years

**DOI:** 10.3389/fmicb.2017.01823

**Published:** 2017-09-20

**Authors:** Rongjun Wang, Guanghui Zhao, Yunya Gong, Longxian Zhang

**Affiliations:** ^1^College of Animal Science and Veterinary Medicine, Henan Agricultural University Zhengzhou, China; ^2^College of Veterinary Medicine, Northwest A&F University Yangling, China; ^3^College of Animal Science and Technology, Henan University of Science and Technology Luoyang, China

**Keywords:** *Cryptosporidium*, cattle, genotype, subtype, population structure, China

## Abstract

Major progress has been made in understanding the epidemiology of bovine *Cryptosporidium* in China in the past 30 years. The overall infection rate in that period was 14.50% (5265/36316), with different prevalence being observed among dairy cattle, yaks, beef cattle, and buffalo. The infection rate declined as the animals’ ages increased and the lowest prevalence occurred in winter. Ten *Cryptosporidium* species and two genotypes have been found in cattle, with *Cryptosporidium parvum*, *C. andersoni*, *C. bovis*, and *C. ryanae* being the commonest species. *Cryptosporidium bovis* rather than *C. parvum* predominated in preweaned dairy cattle, and *C. parvum* IIdA15G1 and IIdA19G1 were the only subtypes detected in dairy cattle. Two subtype families, IIa and IId, were found in yaks. Population genetic analysis detected an epidemic population structure in *C. andersoni*, which suggested that the prevalence of *C. andersoni* in China is not attributable to the introduction of dairy cattle. Moreover, *C. parvum* IId subtypes probably dispersed from western Asia to other geographic regions based on population genetic analysis of isolates from China, Sweden, and Egypt. Therefore, we hypothesize that *Cryptosporidium* was introduced into China in the past, and different populations formed progressively in various hosts in response to diverse factors, including the transmission dynamics, geographic isolation, host specificity, and large-scale farming. More epidemiological studies are required to test this hypothesis and to clarify the prevalence and transmission of *Cryptosporidium* species in China.

## Introduction

*Cryptosporidium* spp. are important zoonotic agents infecting a wide spectrum of vertebrate hosts (Xiao et al., 2004; Wang et al., 2011b). There is extensive genetic variation within the genus *Cryptosporidium*. To date, thirty-one recognized species and more than 60 *Cryptosporidium* genotypes have been discovered (Xiao et al., 2004; Wang et al., 2010; Ryan et al., 2014). Recent studies into the global causes of severe diarrhea in children suggested that *Cryptosporidium* is the second most important diarrheal pathogen after *Rotavirus* (Kotloff et al., 2013; Checkley et al., 2015; Vinayak et al., 2015).

Members of the genus *Cryptosporidium* complete all developmental stages in a single host. Sporulated oocysts, containing four sporozoites, are released from an infected host upon defecation (O’Hara and Chen, 2011). Following ingestion by a suitable host, the motile, infective sporozoites are released through a suture in the oocyst wall and parasitize epithelial cells of the gastrointestinal tract or other tissues (Reduker et al., 1985; O’Hara and Chen, 2011). In these cells, the parasites undergo asexual multiplication, which produces Type-I and Type-II merozoites (Current and Reese, 1986). Type-II merozoites ultimately produce either male or female equivalent sexual reproductive stages, microgametocytes and macrogametocytes, respectively (O’Hara and Chen, 2011). After the macrogamonts are fertilized by the microgametes, oocysts are formed and then sporulated in the infected host. Thick-walled and thin-walled representing two different types of oocysts are produced. The former is commonly excreted from the host, and the latter is primarily involved in autoinfection. *Cryptosporidium* can be transmitted by the fecal-oral route, via either direct contact or ingestion of contaminated food or water (Xiao et al., 2004; Wang et al., 2011c).

Cattle are the mammals in which *Cryptosporidium* infection is most commonly found, and preweaned calves are considered the most important reservoir for zoonotic infection (Wang et al., 2011b; Li F. et al., 2016). *Cryptosporidium parvum*, *C. bovis*, *C. andersoni*, and *C. ryanae* are predominantly responsible for bovine cryptosporidiosis, although several other *Cryptosporidium* species and genotypes are also discovered in cattle, including *C. felis*, *C. hominis*, *C. suis*, *C. scrofarum*, *C. meleagridis*, and *C. suis*-like genotype (Trout and Santín, 2008; Wang et al., 2011b; Zhang et al., 2013; Huang et al., 2014; Robertson et al., 2014). Studies conducted in numerous industrialized nations have suggested that *C. parvum* is the species most often found in preweaned calves and that it is a significant cause of diarrhea (Wang et al., 2011b). *Cryptosporidium bovis* and *C. ryanae* usually infect weaned calves and yearlings, although *C. bovis* is more commonly seen than *C. ryanae*, but neither are associated with the occurrence of diarrhea (Santín et al., 2008; Wang et al., 2011b). In contrast, *C. andersoni* is commonly observed in adult cattle and has been associated with gastritis, reduced milk yield, and poor weight gain (Esteban and Anderson, 1995; Wang et al., 2011b).

*Cryptosporidium* infections are frequently detected in humans and various domestic and wild animals in China (Wang et al., 2008, 2010, 2011c, 2014a; Feng et al., 2012). Among these, cattle are one of the major targets in which *Cryptosporidium* is studied. To date, 97 papers involving 24 provinces, autonomous districts, and municipalities of China have been published on *Cryptosporidium* infections in cattle since the first case was reported in Zhou et al. (1985). The present paper focuses on the advances in the molecular epidemiology of bovine *Cryptosporidium* that have occurred in China in the past 30 years.

## *Cryptosporidium* Infection Rate

These data were calculated from 97 published papers reporting bovine *Cryptosporidium* infections in China. The overall infection rate was 14.50% (5265/36316), with a prevalence of 13.98% (4405/31504), 20.92% (667/3189), 10.47% (122/1165), and 15.50% (71/458) in dairy cattle, yaks, beef cattle, and buffalo, respectively (χ^2^ = 128.32; *P* < 0.01). The infection rate for *Cryptosporidium* species was 45.78% (141/308) in diarrheal calves (Zhou et al., 1985; Chen et al., 1992; Qin et al., 1994; Cui et al., 2014), which was significantly higher than the average prevalence in cattle (14.50%). A correlation between the prevalence and the age of the animals was observed. In general, the infection rate declined as the age of the animals increased (Guo et al., 1993; Chen et al., 2011; Wang et al., 2011a,b; Mi et al., 2013; Ma et al., 2014). In dairy cattle in Henan Province, the infection rates of *Cryptosporidium* species were 21.5% (172/801) in preweaned calves, 11.3% (86/758) in 3–11-month-old calves, 5.7% (5/262) in 12–24-month-old heifers, and 1.0% (3/295) in >24-month-old adult cattle (Wang et al., 2011a,b). In contrast, only two studies have determined the prevalence of *Cryptosporidium* across different seasons, and the prevalence was lowest in winter in both dairy cattle (Wang et al., 2011b) and yaks (Mi et al., 2013).

## *Cryptosporidium* Species Distribution

A total of 1690 *Cryptosporidium*-positive isolates were genotyped and ten *Cryptosporidium* species and two genotypes were identified, including *C. bovis*, *C. andersoni*, *C. ryanae*, *C. parvum*, *C. xiaoi*, *C. ubiquitum*, *C. meleagridis*, *C. hominis*, *C. tyzzeri*, *C. serpentis*, *C. suis*-like genotype, and a new genotype (**Table 1**). *Cryptosporidium bovis* (129/364) rather than *C. parvum* (119/364) was the predominant *Cryptosporidium* species in preweaned dairy cattle (Wang et al., 2011b; Zhang et al., 2013; Cui et al., 2014; Huang et al., 2014; Qi et al., 2015). The earliest detection of *C. bovis* was in 1-week-old calves, indicating that the prepatent period was shorter than the previously recorded 10–12 days (Wang et al., 2011b). *Cryptosporidium andersoni* was most commonly found in heifers and adult cattle (Wang et al., 2011a). In contrast, another two common species, *C. bovis* and *C. ryanae*, occurred at different rates in different epidemiology studies. It is noteworthy that *C. tyzzeri* (formerly *Cryptosporidium* mouse genotype I) and *C. serpentis* probably arose from contamination (Chen and Huang, 2012), and the authors stated in the GenBank submissions that identical sequences (DQ855266 and DQ855267) were found in isolates from pigs with a reverse transcription–PCR analysis of the small subunit ribosomal RNA (Chen and Huang, 2007), and several SSU rRNA gene sequences of *C. tyzzeri* (EU369382, EF025503, EU369384, EU369381, and EU369383) previously isolated from bovine samples have also been deposited in GenBank. Neither *C. tyzzeri* nor *C. serpentis* is a known bovine parasite (Wang et al., 2011a,b).

**Table 1:** *Cryptosporidium* species/genotypes identified in dairy cattle, yaks, beef cattle, and buffalo in China.

Animal	Isolate no.	*Cryptosporidium* species/genotype	Reference
Dairy cattle	1437	*C. andersoni* (457), *C. parvum* (315), *C. bovis* (332), *C. ryanae* (86), *C. tyzzeri* (185), *C. serpentis* (4), *C. hominis* (24), *C. meleagridis* (5), *C. bovis* + *C. ryanae* (9), *C. parvum* + *C. bovis* (6), *C. parvum* + *C. ryanae* (4), *C. parvum* + *C. andersoni* (3)	Watanabe et al., 2005; Feng et al., 2007; Zhou et al., 2007; Liu et al., 2009; Su et al., 2011; Wang et al., 2011a,b; Chen and Huang, 2012; Zhang et al., 2013, 2015; Cui et al., 2014; Huang et al., 2014; Ma et al., 2015; Qi et al., 2015, 2016
Yak	337	*C. andersoni* (75), *C. parvum* (28), *C. bovis* (143), *C. ryanae* (78), *C. ubiquitum* (2), *C. xiaoi* (1), *C. suis*-like genotype (2), *C. parvum* + *C. bovis* (2), *C. bovis* + *C. ryanae* (4), new genotype (2)	Mi et al., 2013; Ma et al., 2014; Qin et al., 2014; Qi et al., 2015; Li P. et al., 2016
Beef cattle	108	*C. andersoni* (85), *C. bovis* (16), *C. ryanae* (6), *C. bovis* + *C. ryanae* (1)	Ma et al., 2015; Qi et al., 2016
Buffalo	40	*C. bovis* (7), *C. ryanae* (33)	Ma et al., 2015


As in dairy cattle, *C. parvum*, *C. andersoni*, *C. bovis*, and *C. ryanae* were also the four *Cryptosporidium* species most commonly identified in yaks, although several other *Cryptosporidium* species and genotypes have been detected (**Table 1**). However, the distribution of different *Cryptosporidium* species according to yak age is still unclear. A study conducted in Qinghai suggested that *C. bovis* was the predominant species in yaks ≤ 2 years old, whereas *C. parvum* was more common in older yaks (Mi et al., 2013). In contrast, a recent study in Tibet showed that *C. andersoni* was predominant in 1–2-year-old yaks, whereas *C. bovis* was commonly found in older yaks (Li P. et al., 2016). Therefore, more studies are required to clarify the *Cryptosporidium* distributions according to age in different groups of animals. Two studies that genotyped *Cryptosporidium*-positive isolates from beef cattle and buffalo identified only *C. andersoni*, *C. bovis*, and *C. ryanae* (Ma et al., 2015; Qi et al., 2016).

## *Cryptosporidium* Subtypes

Subtyping tools have been used extensively in studies of the transmission of *C. hominis*, *C. parvum*, and several other related *Cryptosporidium* species, including *C. meleagridis* and *C. ubiquitum*, in both humans and animals (Xiao, 2010; Li et al., 2014; Ryan et al., 2014). One of the most frequently used subtyping tools is a DNA sequence analysis of the 60-kDa glycoprotein (gp60, also known as gp40/15) gene (Ryan et al., 2014).

In dairy cattle, a total of 141 *C. parvum* isolates have been subtyped by sequencing of the gp60 gene (**Table 2**). Only the IId subtype family was identified, including IIdA15G1 in Gansu Province and the Ningxia Hui Autonomous Region (Cui et al., 2014; Huang et al., 2014; Zhang et al., 2015) and IIdA19G1 in Henan and Heilongjiang Provinces (Wang et al., 2011b; Zhang et al., 2013). Another study described a cryptosporidiosis outbreak caused by *C. parvum* subtype IIdA15G1 on a dairy farm in northwestern China (Cui et al., 2014). Three *C. meleagridis* isolates from preweaned calves were subtyped as IIIeA22G2R1, which was not identical to any known *C. meleagridis* subtype (Zhang et al., 2013). The subtypes of *C. parvum* in yaks appear to be more heterogeneous than those in dairy cattle. Thirteen *Cryptosporidium*-positive isolates from yaks in Qinghai Province were identified as family IIa and five subtypes were detected (Mi et al., 2013). In another study, three IId subtypes (IIdA15G1, IIdA18G1, and IIdA19G1) were detected in five yaks from Qinghai, Gansu, and Tibet, and one *C. ubiquitum* isolate belonged to zoonotic XIIa subtype 2 (Qi et al., 2015). To date, both *C. parvum* subtype IIdA19G1 and *C. ubiquitum* XIIa subtype 2 have also been found in humans in China (Wang et al., 2013; Li et al., 2014).

**Table 2:** *Cryptosporidium* subtypes identified in dairy cattle and yaks in China.

Animal	*Cryptosporidium* species	Subtypes	Reference
Dairy cattle	*C. parvum*	IIdA15G1 (86), IIdA19G1 (55)	Wang et al., 2011b; Zhang et al., 2013, 2015; Cui et al., 2014; Huang et al., 2014
	*C. meleagridis*	IIIeA22G2R1 (3)	Zhang et al., 2013
Yak	*C. parvum*	IIdA15G1 (3), IIdA18G1 (1), IIdA19G1 (1), IIaA15G2R1 (8), IIaA16G2R1 (2), IIaA14G1R1 (1), IIaA14G2R1 (1), IIaA16G3R1 (1)	Mi et al., 2013; Qi et al., 2015
	*C. ubiquitum*	XIIa subtype 2 (1)	Qi et al., 2015


## Population Genetics

**FIGURE 1:**
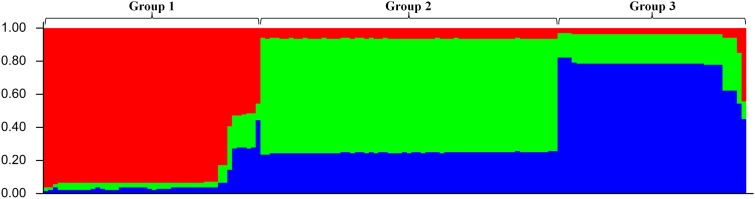
Population structure inferred by Bayesian clustering using multilocus subtype informations of *C. andersoni*. K-means partitional clustering and the admixture mode were used in STRUCTURE 2.3.4 and the most appropriate number of K was calculated using an *ad hoc* statistic-based approach implemented in Structure Harvester v0.6.94 (http://taylor0.biology.ucla.edu/struct_harvest/).

With the development of *Cryptosporidium* subtyping tools, it has become possible to assess the genetic and population structures of *Cryptosporidium* species (Xiao and Ryan, 2008). A total of 149 *C. andersoni* isolates from beef cattle (*n* = 38) and dairy cattle (*n* = 111) were subtyped with a multilocus sequence typing (MLST) tool based on the MS1, MS2, MS3, and MS16 loci. Fourteen MLST subtypes were identified and A4,A4,A4,A1 was the predominant subtype (Wang et al., 2012; Zhao et al., 2013; Qi et al., 2016). To test the possibility that this linkage disequilibrium (LD) was attributable to the clonal expansion of one or more subtype, masking the underlying equilibrium, an LD analysis was conducted using only the MLST subtypes (by considering each group of isolates with the same MLST subtype as one individual (Wang et al., 2012), 14 MLST subtypes were used in this analysis), with LIAN version 3.7. This analysis suggested that the *C. andersoni* population in cattle in China had an epidemic population structure (*I*^S^_A_ = 0.0010, *V*_D_ < *L*). An analysis using STRUCTURE 2.3.4 with K-means partitional clustering and the admixture model revealed three ancient lineages among the 149 *C. andersoni* specimens (**Figure 1**).

*Cryptosporidium parvum* is another species that has been targeted for the population genetic analysis in cattle. *Cryptosporidium parvum* IId isolates (*n* = 111) from several species of animals in China, Sweden, and Egypt (Wang et al., 2014b) were subtyped with an MLST tool based on 12 microsatellite, minisatellite, and single-nucleotide polymorphism loci (Wang et al., 2014b). Host adaptation and significant geographic segregation were both observed in the MLST subtypes. A clonal population structure was detected in the *C. parvum* IId isolates from China and Sweden. Three ancestral lineages and the same RPGR (retinitis pigmentosa GTPase regulator) sequence were shared by the isolates examined (Wang et al., 2014b). The authors concluded that the *C. parvum* IId subtypes probably dispersed from western Asia to other geographic regions (Wang et al., 2014b; Zahedi et al., 2016).

## Conclusion and Perspectives

Epidemiological data suggest that *Cryptosporidium* infections are commonly found in cattle in China. The infection rate declines as the age of the animals increases, and the lowest infection rate occurs in winter. Similar to the species distributions reported in other countries and areas of the world, *C. parvum*, *C. bovis*, *C. andersoni* and *C. ryanae* are the four commonest *Cryptosporidium* species in cattle. *Cryptosporidium bovis* rather than *C. parvum* was the dominant *Cryptosporidium* species in preweaned dairy cattle, which differs from the dominant species in this age group in other countries and areas of the world. Uniquely, the *C. parvum* subtypes identified in dairy cattle were all zoonotic IIdA15G1 or IIdA19G1 (Wang et al., 2014b).

Population genetic analyses of 149 *C. andersoni* isolates in three published studies confirmed an epidemic population structure, and as proposed in a previous study, these data suggest that the prevalence of *C. andersoni* in China is not attributable to the introduction of dairy cattle (Wang et al., 2012). According to a population genetic analysis, the *C. parvum* IId subtypes probably dispersed from western Asia to other geographic regions (Wang et al., 2014b). Therefore, we hypothesize that *Cryptosporidium* was introduced into China at some time in the past, and then different *Cryptosporidium* populations developed progressively in various hosts in response to diverse factors, including the transmission dynamics, geographic isolation, host specificity, and large-scale farming. More epidemiological studies are required to confirm this hypothesis and to clarify the transmission and public-health impact of *Cryptosporidium* species in China.

## Author Contributions

LZ had the ideal for the review and revised the manuscript. RW wrote the paper. RW, GZ, and YG reviewed and abstracted data from each selected article.

## Conflict of Interest Statement

The authors declare that the research was conducted in the absence of any commercial or financial relationships that could be construed as a potential conflict of interest.
